# Overlapping Genetic Architecture Between Schizophrenia and Neurodegenerative Disorders

**DOI:** 10.3389/fcell.2021.797072

**Published:** 2021-12-24

**Authors:** Chunyu Li, Tianmi Yang, Ruwei Ou, Huifang Shang

**Affiliations:** Department of Neurology, Laboratory of Neurodegenerative Disorders, National Clinical Research Center for Geriatrics, West China Hospital, Sichuan University, Chengdu, China

**Keywords:** TWAS, genetic correlation, schizophrenia, neurodegeneration, expression profiles

## Abstract

Epidemiological and clinical studies have suggested comorbidity between schizophrenia and several neurodegenerative disorders. However, little is known whether there exists shared genetic architecture. To explore their relationship from a genetic and transcriptomic perspective, we applied polygenic and linkage disequilibrium-informed methods to examine the genetic correlation between schizophrenia and amyotrophic lateral sclerosis (ALS), Parkinson’s disease, Alzheimer’s disease and frontotemporal dementia. We further combined genome-wide association summary statistics with large-scale transcriptomic datasets, to identify putative shared genes and explore related pathological tissues. We identified positive and significant correlation between schizophrenia and ALS at genetic (correlation 0.22; 95% CI: 0.16–0.28; *p* = 4.00E-04) and transcriptomic (correlation 0.08; 95% CI: 0.04–0.11; *p* = 0.034) levels. We further demonstrated that schizophrenia- and ALS-inferred gene expression overlap significantly in four tissues including skin, small intestine, brain cortex and lung, and highlighted three genes, namely *GLB1L3*, *ZNHIT3* and *TMEM194A* as potential mediators of the correlation between schizophrenia and ALS. Our findings revealed overlapped gene expression profiles in specific tissues between schizophrenia and ALS, and identified novel potential shared genes. These results provided a better understanding for the pleiotropy of schizophrenia, and paved way for future studies to further elucidate the molecular drivers of schizophrenia.

## Introduction

Schizophrenia is a chronic and severe mental disorder characterized by continuous or relapsing episodes of psychosis with major symptoms like hallucinations and delusions (Owen et al., 2016). Usually, schizophrenia is considered as a neurodevelopmental disorder attributed to alterations in prenatal-to-early adolescent development. However, emerging evidence from neuropathological and longitudinal studies of schizophrenia also supports a neurodegenerative hypothesis. For example, schizophrenia was suggested as associated with accumulated protein insolubility and ubiquitination in a way similar to that presented in cases of neurodegenerative diseases (Nucifora et al., 2019). Meanwhile, abnormal white matter (WM) integrity as indicated by reduced fractional anisotropy at the onset and during chronic states of schizophrenia was observed in patients compared with controls, and the difference might increase over time (Kochunov and Hong, 2014). Mapping the trajectory of WM changes along the lifespan could provide novel insight into the biological nature of the factors underlying WM deficits (Cetin-Karayumak et al., 2020). The accelerated decline with aging also supported the neurodegenerative model. Even though, such a hypothesis was still debated due to various factors such as the absence of characteristics of neurodegeneration in schizophrenia, like gliosis (Gupta and Kulhara, 2010). Therefore, the link between schizophrenia and neurodegeneration might be complex and worth further exploration.

Clinically, growing evidence suggested that schizophrenia was linked to several neurodegenerative diseases. Amyotrophic lateral sclerosis (ALS), classically described as a motor system disorder, has been reported to co-exist with a variety of mental symptoms like psychosis and dementia, while disturbances in motor neuron function at both central and peripheral levels existed in schizophrenia (Crayton et al., 1977; Crayton and Meltzer, 1979; Zucchi et al., 2019). Meanwhile, a recent genetic study identified genetic pleiotropy between ALS and schizophrenia using the conditional false discovery rate method (McLaughlin et al., 2017). Both schizophrenia and Parkinson’s disease (PD) were closely related to dopaminergic function, with dopaminergic system imbalance or dysfunction as a common cause (Salamone et al., 2016). In clinical practice, several cases with both schizophrenia and PD have been reported, suggesting potential commodities between the two disorders (Habermeyer et al., 2008; Gadit, 2011). Psychotic symptoms like delusions and hallucinations which were characteristics of schizophrenia, were also frequent in Alzheimer’s disease (AD), affecting about 40–60% of individuals with AD. And AD with psychosis was found inversely associated with polygenic risk for schizophrenia (DeMichele-Sweet et al., 2018; Creese et al., 2019). Frontotemporal dementia (FTD) is a disorder of frontal lobe function, while recent research has demonstrated changes in the frontal and temporal lobes in schizophrenia as well. Meanwhile, schizophrenia and FTD patients presented with some similar clinical symptoms, and molecular evidence has been proposed for potential shared pathophysiologic mechanisms (Cooper and Ovsiew, 2013). Taken together, schizophrenia was closely correlated with these neurodegenerative disorders and might share common pathogenesis with them.

Recently, the integration of large-scale functional genomic data with genome-wide association study (GWAS) results has been proposed to characterize the functional effects of associated variants (Li et al., 2019). Transcriptome-wide association study (TWAS) is a powerful approach to research the genetic architecture of complex traits like schizophrenia, by building a model to impute gene expression levels from genotypes using samples with matched genotypes and gene expression data in given tissues (Hu et al., 2019). In this context, we explored the genetic correlation between schizophrenia and four neurodegenerative diseases including ALS, PD, AD, and FTD, and identified a positive and significant correlation between schizophrenia and ALS. We further conducted transcriptome-wide expression analysis and identified specific tissues and genes with overlapping expression profiles between schizophrenia and ALS.

## Methods

### GWAS Summary Statistics

We investigated the genetic correlation between schizophrenia (Lam et al., 2019) and four neurodegenerative disorders, namely ALS (Nicolas et al., 2018), PD (Nalls et al., 2019), AD (Lambert et al., 2013) and FTD (Ferrari et al., 2014) based on GWAS summary statistics. Details of the summary data from all GWAS used in the current study were listed in Supplementary Table S1. The study design like the collection of samples, quality control procedures and imputation methods have been described in each publication. The relevant institutional review boards or ethics committees approved the research protocol of each GWAS, and all participants gave written informed consent.

### Transcriptomic Atlases for LDSC-SEG

We first applied linkage disequilibrium (LD) score regression in specifically expressed genes (LDSC-SEG) to identify disease-relevant tissues and cell types by analyzing gene expression data together with GWAS summary statistics (Finucane et al., 2018). LDSC-SEG identifies tissues in which genes with increased expression are enriched in single-nucleotide polymorphisms (SNPs) that tag a large amount of heritability. Data for LDSC-SEG was prepared as described earlier (Finucane et al., 2018). Gene expression data from the following sources were analyzed: 1) RNA sequencing (RNA-seq) read counts from Genotype-Tissue Expression (GTEx) v6p (Gamazon et al., 2018). Genes for which fewer than four samples had at least one read count per million, as well as samples for which fewer than 100 genes had at least one read count per million were removed. 2) GeneChip expression array data from mouse forebrain sorted cells downloaded in the Gene Expression Omnibus (GEO) (GSE9566) (Cahoy et al., 2008). 3) Publicly available gene expression data from the ImmGen project (Heng and Painter, 2008) downloaded in GEO (GSE15907, GSE37448). A *p* value below 9.43E-04 (0.05/53), 0.017 (0.05/3), and 1.71E-04 (0.05/292) was considered statistically significant after the Bonferroni correction for analysis using the GTEx, GSE9566 and ImmGen data respectively.

### Genetic Correlation

We estimated the genetic correlation between schizophrenia and each neurodegenerative disorder using LDSC (Bulik-Sullivan et al., 2015) and GNOVA (Lu et al., 2017) with default parameters. The LDSC method quantifies the contribution of each SNP by examining the relationship between GWAS summary statistics and linkage disequilibrium (Abecasis et al., 2012). Compared with LDSC, GNOVA corrects for sample overlap and provides greater statistical power as well as higher estimation accuracy. We ran LDSC and GNOVA on SNPs in both diseases together with reference data derived from the 1,000 Genomes Project European population. Genetic correlation and *p* values were corrected for sample overlap for GNOVA. A *p* value below 0.0025 (0.05/20) was considered statistically significant after the Bonferroni correction.

### Gene Expression Overlap

We investigated whether the genetic overlap between schizophrenia and each neurodegenerative disease was mediated by shared regulation of gene expression. We generated tissue-specific, disease-inferred gene expression profiles using TWAS software with default parameters (Gusev et al., 2016). TWAS integrates gene expression measurements with GWAS summary statistics to identify genes whose cis-regulated expression is associated with complex traits (Gusev et al., 2016). Briefly, TWAS leverages large-scale RNA-seq data to impute tissue-specific gene expression levels from GWAS summary statistics, which can further be used to identify novel associated genes. RNA-seq data from 48 tissues in the GTEx v7 reference panel (Battle et al., 2017) were used to generate all disease-inferred gene expression profiles. Summary statistics of all the SNPs from each GWAS were utilized. Furthermore, we estimated the overlap between the disease-inferred gene expression with RHOGE (Mancuso et al., 2017), using TWAS results with nominal association (*p* < 0.05). RHOGE estimates the genetic correlation between two traits which can be attributed to cis-expression quantitative trait loci (eQTL) as represented by different trait-inferred gene expression profiles. The major histocompatibility complex (MHC) region was excluded due to its complex LD structure. A *p* value below 0.0010 (0.05/48) was considered statistically significant after the Bonferroni correction.

### Genes Underlying Disease-Inferred Gene Expression Overlap

We used UTMOST to infer the genes for schizophrenia and ALS underlying the overlapping disease-inferred gene expression across tissues (Hu et al., 2019). UTMOST combines multiple single-tissue associations into a powerful metric to quantify the overall gene-trait association and is suggested to achieve improved accuracy than single-tissue strategy. First, we generated the single-tissue expression model using GWAS summary statistics and matched expression data from all tissues in GTEx with default parameters. Then we performed a cross-tissue test for each gene to summarize single-tissue association statistics to quantify the overall gene-trait association. We identified transcriptome-wide significant gene-disease associations using a false discovery rate (FDR) threshold of 0.001.

### Differential Expression in Patients and Controls

To determine whether the identified risk genes were differentially expressed in tissues from patients with ALS or schizophrenia compared with healthy controls, we analyzed the gene expression of the target genes in whole blood from 233 ALS patients and 508 controls (GEO accession number GSE8397, GPL6947), and neurons from human induced pluripotent stem cell (hiPSC) in 12 schizophrenia patients and 12 controls (GEO accession number GSE25673). Data were prepared and analyzed using GEO2R (Barrett et al., 2013), and a *p* value below 0.05 was considered significant after adjusting for multiple testing.

## Results

### Transcriptomic Atlases for LDSC-SEG

**FIGURE 1:**
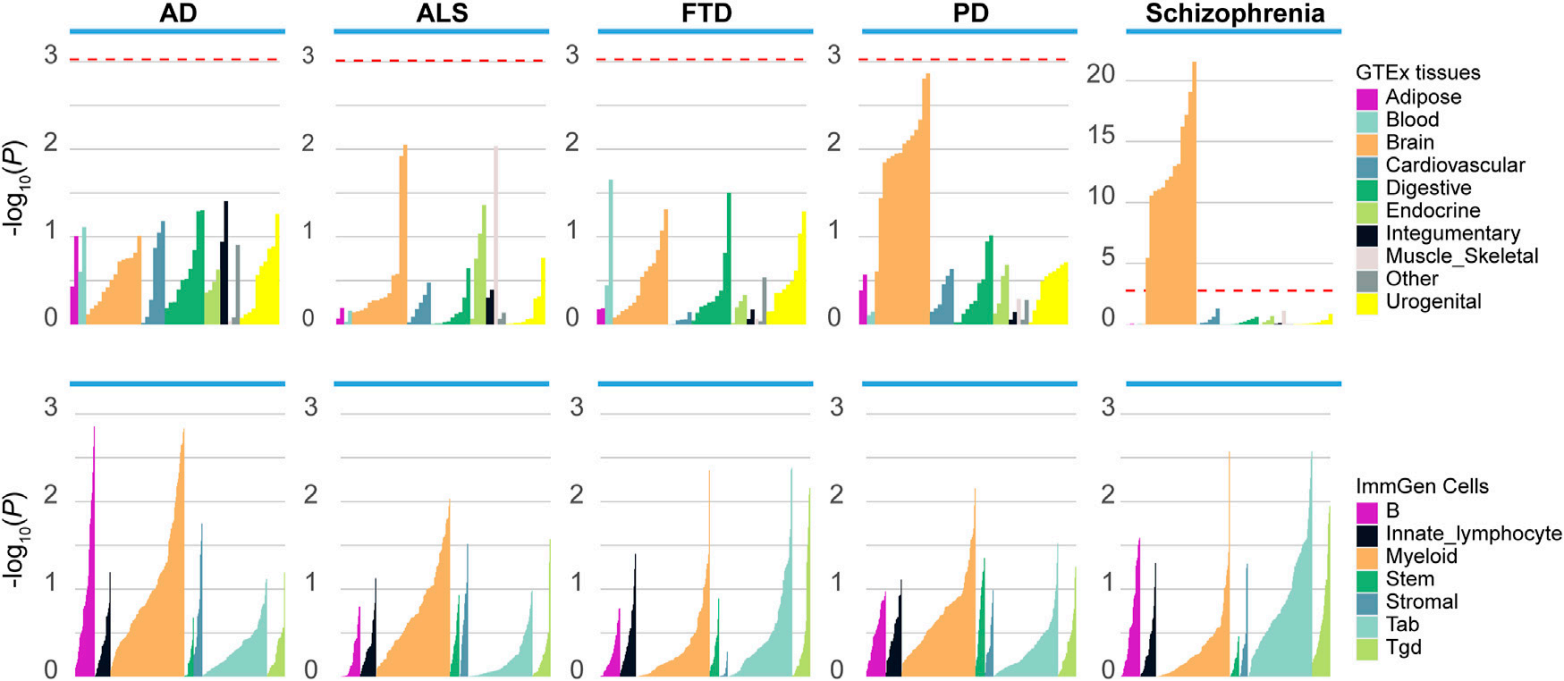
Enrichment of tissues and cell types in schizophrenia and neurodegenerative diseases. Top panel: Regions of the genome with specific expression in central nervous system (CNS) tissues are highly enriched for schizophrenia and PD, among 53 tissues obtained from the Genotype-Tissue expression project (GTEx). Bottom panel: LDSC-SEG analysis using 292 immune cells from the ImmGen Consortium. ALS, amyotrophic lateral sclerosis; PD, Parkinson’s disease; AD, Alzheimer’s disease; FTD, frontotemporal dementia. The red dashed line denotes the significance level after the Bonferroni correction.

To better understand how the genetic variants affected disease risk, we used LDSC-SEG to identify tissues or cell types likely to be relevant to the etiology of each disease. When applying to 53 tissues from the GTEx project, we detected significant enrichment in 13 brain-related tissues for schizophrenia, and nominal enrichment in brain-related tissues for PD (Figure 1). In contrast, there is less enrichment of SNP heritability near genes expressed in brain-related tissues for ALS, AD and FTD. Moreover, we applied LDSC-SEG to expression data from sorted primary mouse CNS cells (Cahoy et al., 2008). We found significant enrichment in neuron (*p* = 2.11E-05) for schizophrenia, as well as nominal enrichment in oligodendrocyte (*p* = 0.037) and neuron (*p* = 0.049) for PD (Supplementary Figure S1). Finally, considering that the immune dysfunction was closely related to the pathophysiology of schizophrenia (Müller and Schwarz, 2010), we applied LDSC-SEG on an atlas of 292 mouse immunological cell types (Heng and Painter, 2008) to assess the cell types where signal enrichment was specifically expressed. Though no significant association was identified, a marked enrichment in myeloid cell types was observed for ALS, PD, and AD, while schizophrenia was relatively enriched in T cells (Figure 1). Altogether, these results suggested that schizophrenia was associated with gene expression in brain-related tissues, neurons and T cells, while the other neurodegenerative disorders were less enriched in these tissues and cell types.

### Genetic Correlation

We detected a significant and positive genetic correlation between ALS and schizophrenia using both LDSC (genetic correlation: 0.22; *p* = 4.00E-04) and GNOVA (genetic correlation 0.13, *p* = 6.83E-05) methods after the Bonferroni correction (Table 1). No significant genetic correlation was observed between the other diseases.

**TABLE 1:** Genetic correlation between schizophrenia and four neurodegenerative disorders.

Trait	Schizophrenia	AD	ALS	FTD	PD
schizophrenia	—	0.05 (0.37)	**0.22 (4.00E-04)**	0.23 (0.07)	0.02 (0.48)
AD	0.05 (0.04)	—	0.14 (0.46)	0.24 (0.39)	0.01 (0.90)
ALS	**0.13 (6.83E-05)**	0.11 (0.05)	—	0.25 (0.51)	0.11 (0.37)
FTD	0.21 (0.01)	0.22 (0.11)	0.07 (0.72)	—	0.51 (0.02)
PD	−0.02 (0.33)	0.06 (0.18)	0.06 (0.27)	0.42 (3.18E-03)	—

AD, Alzheimer’s disease; ALS, amyotrophic lateral sclerosis; FTD, frontotemporal dementia; PD, Parkinson’s disease. *p* values in bold denotes significant associations. Values in the right-upper side are calculated using LDSC, while values in the left-lower side are calculated using GNOVA. *p* values are presented in parentheses.

### Schizophrenia and ALS Gene Expression Overlaps Across Tissues

**FIGURE 2:**
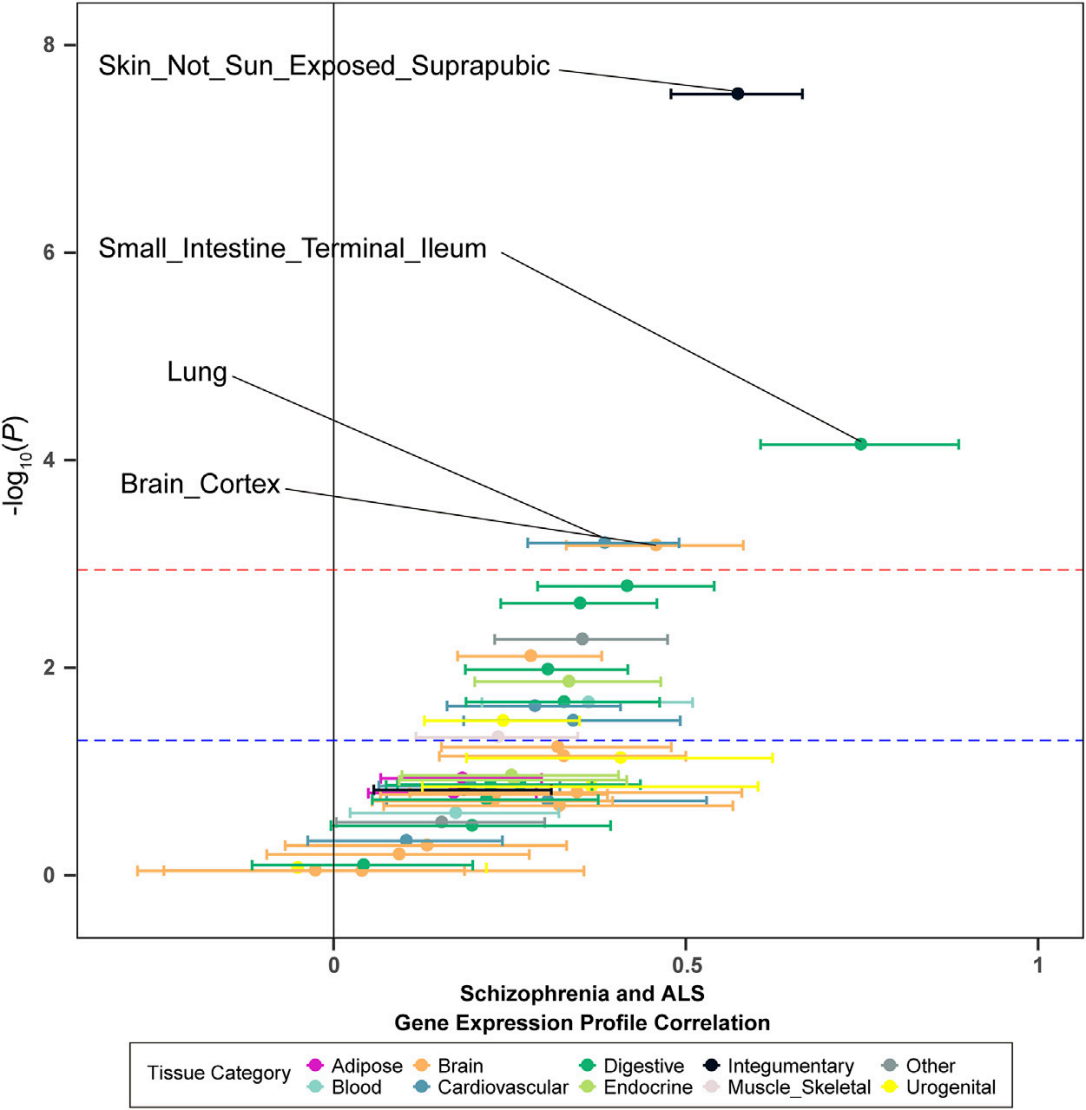
Tissue-specific disease-inferred gene expression profile correlation between schizophrenia and ALS. The red dashed line demarks the multiple-test corrected threshold (*p* = 0.05/48), while the blue dotted line demarks the nominal threshold (*p* = 0.05).

To investigate whether the genetic overlap between schizophrenia and ALS was mediated by shared regulation of gene expression, we generated tissue-specific gene expression profiles using TWAS, and further estimated the overlap between the different disease-inferred gene expression profiles using RHOGE. We identified a positive overlap between ALS- and schizophrenia-inferred gene expression profiles in a joint analysis of the GTEx v7 reference panel (gene expression correlation: 0.08; 95% CI: 0.04–0.11; *p* = 0.034). Analyzing the genetic correlation in each tissue separately, we observed positive and significant overlap in not-sun-exposed skin (gene expression correlation: 0.57, 95% CI: 0.48–0.67; *p* = 2.97E-08), small intestine tissues (gene expression correlation: 0.75; 95% CI: 0.61–0.89; *p* = 7.07E-05), lung (gene expression correlation: 0.38; 95% CI: 0.28–0.49; *p* = 6.29E-04) and brain cortex (gene expression correlation: 0.46; 95% CI: 0.33–0.58; *p* = 6.63E-04) (Figure 2; Table 2). The overall expression overlap was even more significant between schizophrenia and ALS when considering only these four tissues together (gene expression correlation: 0.39; 95% CI: 0.32–0.46; *p* = 2.42E-07). For the other three neurodegenerative disorders, only skeletal muscle was found with overlapped expression profiles between FTD and schizophrenia (Supplementary Figures S2–S4; Supplementary Table S2).

**TABLE 2:** Disease-inferred gene expression profile overlap between ALS and schizophrenia in GTEx v7 reference panel tissues.

GTEx tissue	Correlation (95% CI)	*p* Value
Adipose_Subcutaneous	0.17 (0.05, 0.29)	1.61E-01
Adipose_Visceral_Omentum	0.18 (0.07, 0.30)	1.16E-01
Adrenal_Gland	0.25 (0.10, 0.40)	1.09E-01
Artery_Aorta	0.19 (0.06, 0.32)	1.38E-01
Artery_Coronary	0.30 (0.08, 0.53)	1.93E-01
Artery_Tibial	0.28 (0.16, 0.41)	2.34E-02
Brain_Amygdala	0.34 (0.11, 0.58)	1.59E-01
Brain_Anterior_cingulate_cortex_BA24	−0.03 (−0.24, 0.19)	8.98E-01
Brain_Caudate_basal_ganglia	0.23 (0.07, 0.39)	1.66E-01
Brain_Cerebellar_Hemisphere	0.32 (0.15, 0.48)	5.81E-02
Brain_Cerebellum	0.23 (0.05, 0.40)	1.93E-01
Brain_Cortex	0.46 (0.33, 0.58)	**6.63E-04**
Brain_Frontal_Cortex_BA9	0.13 (−0.07, 0.33)	5.16E-01
Brain_Hippocampus	0.04 (−0.28, 0.36)	9.05E-01
Brain_Hypothalamus	0.32 (0.07, 0.57)	2.14E-01
Brain_Nucleus_accumbens_basal_ganglia	0.32 (0.15, 0.50)	7.11E-02
Brain_Putamen_basal_ganglia	0.09 (−0.10, 0.28)	6.27E-01
Breast_Mammary_Tissue	0.15 (0.00, 0.30)	3.10E-01
Cells_EBV-transformed_lymphocytes	0.36 (0.21, 0.51)	2.16E-02
Cells_Transformed_fibroblasts	0.35 (0.23, 0.47)	5.32E-03
Colon_Sigmoid	0.33 (0.19, 0.46)	2.14E-02
Colon_Transverse	0.22 (0.07, 0.37)	1.36E-01
Esophagus_Gastroesophageal_Junction	0.26 (0.09, 0.44)	1.34E-01
Esophagus_Mucosa	0.30 (0.19, 0.42)	1.04E-02
Esophagus_Muscularis	0.35 (0.24, 0.46)	2.40E-03
Heart_Atrial_Appendage	0.10 (−0.04, 0.24)	4.66E-01
Heart_Left_Ventricle	0.34 (0.18, 0.49)	3.24E-02
Liver	0.19 (0.00, 0.39)	3.33E-01
Lung	0.38 (0.28, 0.49)	**6.29E-04**
Muscle_Skeletal	0.23 (0.12, 0.35)	4.67E-02
Nerve_Tibial	0.28 (0.18, 0.38)	7.73E-03
Ovary	−0.05 (−0.32, 0.22)	8.46E-01
Pancreas	0.41 (0.29, 0.54)	1.63E-03
Pituitary	0.25 (0.09, 0.42)	1.21E-01
Prostate	0.41 (0.19, 0.62)	7.41E-02
Skin_Not_Sun_Exposed_Suprapubic	0.57 (0.48, 0.67)	**2.97E-08**
Skin_Sun_Exposed_Lower_leg	0.18 (0.06, 0.31)	1.51E-01
Small_Intestine_Terminal_Ileum	0.75 (0.61, 0.89)	**7.07E-05**
Spleen	0.04 (−0.12, 0.20)	7.96E-01
Stomach	0.22 (0.05, 0.38)	1.87E-01
Testis	0.24 (0.13, 0.35)	3.25E-02
Thyroid	0.33 (0.20, 0.46)	1.36E-02
Uterus	0.36 (0.13, 0.60)	1.40E-01
Whole_Blood	0.17 (0.02, 0.32)	2.51E-01

*p* values in bold denotes significant associations.

**FIGURE 3:**
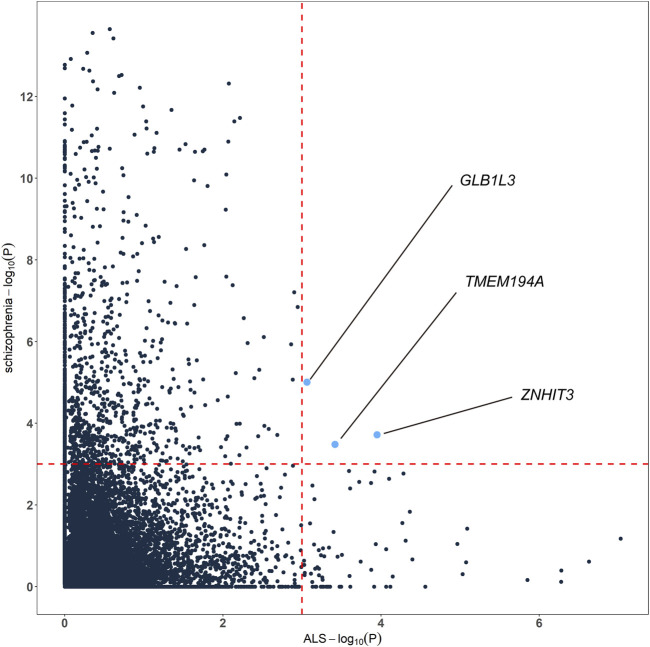
Genes associated with both schizophrenia and ALS cross tissues**.** The red dashed lines denotes the false discovery rate (FDR) threshold of 0.001.

To highlight the genes whose expression was commonly regulated by schizophrenia and ALS risk variants, we generated cross-tissue disease-associated genes using UTMOST. In the results of ALS, known ALS genes like *SCFD1* (*p* = 2.35E-07), *G2E3* (*p* = 5.26E-07), *B4GALNT1* (*p* = 1.41E-06) and so on showed significant association. In the results of schizophrenia, known schizophrenia genes like *WBP1L* (*p* = 2.24E-14), *TMTC1* (*p* = 3.75E-14), *ZNF804A* (*p* = 8.50E-14), *TAOK2* (4.82E-12) and so on showed significant association. Combining the cross-tissue summary metric results, we identified 3 genes passing the FDR threshold for both schizophrenia and ALS, namely *ZNHIT3*, *GLB1L3* and *TMEM194A* (Figure 3; Supplementary Table S3). We further analyzed the expression profiles of the three genes using available expression data from GEO. As a result, we found that *ZNHIT3* (*p* = 1.20E-13) and *TMEM194A* (*p* = 5.12E-07) were differentially expressed between ALS patients and healthy controls in whole blood, and *ZNHIT3* (*p* = 0.02) was differentially expressed in hiPSC-derived neurons between schizophrenia patients and controls.

## Discussion

Leveraging summary statistics from large-scale GWAS, public transcriptomic datasets and the polygenic modeling for complex traits, we investigated the overlapping genetic architecture between schizophrenia and four neurodegenerative disorders. We identified a significant genetic correlation between schizophrenia and ALS, and further located three genes with overlapped gene expression profiles across tissues. Our results provided evidence for shared genetic architecture between schizophrenia and ALS.

Both schizophrenia and neurodegenerative disorders are complex diseases affected by multiple genetic and environmental factors. Previous multi-variant genetic analyses investigating schizophrenia and ALS focused specifically on GWAS summary statistics and thus have missed a substantial proportion of the transcriptome architecture (McLaughlin et al., 2017). By combining GWAS summary statistics and large-scale transcriptome data, we observed a positive correlation between schizophrenia and ALS at both genetic and transcriptomic levels. We further identified overlapped expression profiles in specific tissues including not-sun-exposed skin, small intestine, brain cortex and lung, which were hard to detect using only genetic data. Meanwhile, we also noticed that schizophrenia and PD were both specifically expressed in brain-related tissues using LDSC-SEG, but no genome-wide genetic correlation was found, suggesting different genetic background between PD and schizophrenia. To support this hypothesis, we estimated local SNP heritability using ρ-HESS(Shi et al., 2017), which could estimate and visualize the local SNP heritability from GWAS summary statistics. We could see that the SNP heritability for PD and schizophrenia was enriched in different regions across the chromosomes, suggesting potential diverse genetic architecture (Supplementary Figure S5). In addition, PD symptoms could be alleviated with dopamine receptor agonists, whereas schizophrenia was commonly treated with dopamine receptor antagonists. And dopaminergic treatment used for PD was a particular risk factor for psychoses in PD (Ecker et al., 2009), suggesting differing underlying molecular mechanism.

Although both schizophrenia and ALS mainly affected the central nervous system, growing evidence has indicated that other tissues and organs might be involved in their pathogenesis as well. The brain cortex is primarily responsible for thinking, perceiving and motor activity, and has been shown to play important roles in neurodegenerative and neuropsychiatric disorders. It was noticed that both schizophrenia and ALS patients had a thinner cerebral cortex compared to healthy individuals on average (Walhout et al., 2015; van Erp et al., 2018). Meanwhile, A number ALS risk genes were differentially expressed in motor cortex of ALS patients compared with controls (Aronica et al., 2015). Clinically, both schizophrenia and ALS patients could present cognitive impairment correlated with pathologic and radiographic changes in the cerebral cortex (Woolley and Jonathan, 2008). In the developing embryo, both the brain and skin arise from the same ectodermal germ layer. Researchers have reported that protein aggregates found in the brains of patients with neurodegenerative disorders like ALS, PD and AD appeared in the dermal layers as well (Widmer et al., 2006). Meanwhile, ALS patients have been reported with dermatological conditions such as bullous pemphigoid and decreased collagen, and because of such changes, the skin has been proposed as a source of diagnostic markers for ALS (Watanabe et al., 1987; Kolde et al., 1996; Paré and Gros-Louis, 2017). In addition, increased expression of TDP-43 was found in skin of ALS patients compared with controls (Suzuki et al., 2010). Patients with schizophrenia showed increased rates of skin disorders as well, like psoriasis and atopic dermatitis (McPhie et al., 2020). Previous study found that some messenger RNA and microRNA were differentially expressed between schizophrenia patients and controls, and scalp hair follicles were also suggested as a beneficial genetic biomarker resource for schizophrenia (Maekawa et al., 2015). However, it remains unclear about the exact nature of the association between gene expression changes in skin and the two disorders. Further studies are warranted to elucidate the underlying pathways.

Emerging epidemiological evidence has suggested that intestinal homeostasis and the gut microbiome play essential roles in neurological diseases, and potential function of gut microbiome and metabolites were identified in the ALS model and ALS patients as well (Wu et al., 2015; Blacher et al., 2019; Zeng et al., 2020). Similarly, it was found that patients with schizophrenia had a decreased microbiome α-diversity index and marked disturbances of gut microbial composition versus healthy controls (Zheng et al., 2019). Consistent with these observations, our findings support the involvement of intestinal tissues in the pathogenesis of ALS and schizophrenia, though the mechanism of such relation is still unclear. Recent evidence suggested that the gut microbiota could modulate brain function via the “microbiota-gut-brain” axis (Hsiao et al., 2013; Dinan and Cryan, 2015), and it was also proposed that gastrointestinal function could impact the brain via immune system pathways (Severance et al., 2015). Future explorations are warranted to provide deeper understandings. Besides, we identified lung tissue with overlapped expression profiles. Although patients with schizophrenia or ALS both presented lung dysfunction and respiratory problems (Javad Mousavi et al., 2014), whether they were related to gene expression in lung tissue and how they were correlated has not been explained yet. Previous genetic enrichment analysis between schizophrenia and several cancers identified polygenic enrichment between schizophrenia and lung cancer, and functional analysis identified downstream pleiotropic effects on gene-expression in lung and brain tissues as well (Zuber et al., 2018). Further explorations are necessary to elucidate how lung tissues were involved in schizophrenia.

Beyond the four tissues identified with overlapped expression profiles, we also located three genes underlying the overlapped disease-inferred gene expression across tissues, including *GLB1L3*, *ZNHIT3* and *TMEM194A*. *GLB1L3* was widely expressed in the central nervous system. It was involved in several important metabolic processes such as carbohydrate metabolic process and beta-galactosidase activity, while carbohydrate metabolism has been implicated in both schizophrenia and ALS (Joardar et al., 2017; Bryll et al., 2020). In addition, it was indicated that copy number variants in the *GLB1L3* were potentially associated with schizophrenia (Levinson et al., 2011). *ZNHIT3* encodes a nuclear zinc finger protein which is implicated in transcriptional regulation and metal ion binding activity. Metal ions within the cellular environment were suggested to play a potential role in formation and deposition of toxic protein aggregates in ALS (Sirangelo and Iannuzzi, 2017), and metal ions might also influence the efficacy of treatment of schizophrenia with antipsychotics (Sussulini et al., 2018). In addition, recent summary statistic-based Mendelian randomization (SMR) analysis which combines disease-SNP association and expression-SNP association results identified *ZNHIT3* as a risk gene for ALS (Benyamin et al., 2017; Du et al., 2018). Meanwhile, cellular knockdown of *ZNHIT3* in mouse cerebellar granule cells sensitized the neurons to death, suggesting its potential role in neuron survival (Anttonen et al., 2017). *TMEM194A* is a novel inner nuclear membranes protein expressed across the tissues. We did not find direct evidence for the role of *TMEM194A* in ALS and schizophrenia. Further replication was necessary to explore its potential involvement in the pathogenesis of ALS and schizophrenia.

Our study has several strengthes and limitations worth noting. By combining GWAS summary statistics and large-scale transcriptomic datasets using a TWAS approach, we identified specific tissues with overlapped gene expression profiles between schizophrenia and ALS, as well as three risk genes underlying the correlation. Such method could characterize the functional effects of associated variants for diseases, and get results which cannot be obtained using only genetic data. Moreover, the current results have clinical implications. Since we combined GWAS summary statistics from different but related diseases, the findings may increase our understanding of the pathogenetic mechanisms influenced by pleiotropic genes and facilitate novel treatment strategies in clinical trials. However, the GWASs used in the current study were mostly performed on participants of European ancestry, thus the findings of shared genetic architecture might be biased and not applicable to other populations. Meanwhile, there was a potential sample overlap in the original GWAS. Such overlap might bring some bias to the statistical analysis.

In conclusion, by integrating GWAS summary data and large-scale transcriptomic data, we identified genetic correlation between schizophrenia and ALS. Moreover, we identified four tissues with overlapped expression profiles which might be involved in the pathogenesis of schizophrenia and ALS, and located three novel risk genes underlying the overlapping disease-inferred gene expression across tissues. These findings provided novel insights into the shared genetic background between schizophrenia and neurodegenerative disorders and might help better understand the etiology of schizophrenia.

## Data Availability

The original contributions presented in the study are included in the article/Supplementary Material, further inquiries can be directed to the corresponding authors.

## Abbreviations

AD, Alzheimer’s disease; ALS, amyotrophic lateral sclerosis; eQTL, expression quantitative trait loci; FDR, false discovery rate; FTD, frontotemporal dementia; GWAS, genome-wide association study; LD, linkage disequilibrium; MHC, major histocompatibility complex; PD, Parkinson’s disease; SNP, single nucleotide polymorphism; TWAS, transcriptome-wide association study.
