# Supplementary material for: Overlapping Genetic Architecture Between Schizophrenia and Neurodegenerative Disorders
Source: Front Cell Dev Biol. 2021 Dec 24;9:797072. doi: 10.3389/fcell.2021.797072 (PMC8740133; doi:10.3389/fcell.2021.797072)
Supplement: Supplementary file 1 [file DataSheet1.PDF]

Supplementary Table 1. Summary data from all GWAS used in current study

Disease/trait	abbreviation	Cases	Controls	Ethnics	SNPs	PMID
schizophrenia	schizophrenia	56,418	78,818	EUR & EAS	139,442,226	31740837
amyotrophic lateral sclerosis	ALS	20,806	59,804	EUR	10,031,417	29566793
Parkinson's disease	PD	33,647	449,056	EUR	17,510,617	31701892
Alzheimer's disease	AD	17,008	37,154	EUR	7,055,881	24162737
frontotemporal dementia	FTD	3,526	9,402	EUR	6,026,384	24943344

EUR, European; EAS, East Asian; SNP, single nucleotide polymorphism; GWAS, genome-wide association study; PMID, Pubmed ID

Supplementary Table 2. Disease-inferred gene expression profile overlap between schizophrenia and AD, FTD, PD in GTEx v7 reference panel tissues

GTEx tissue	schizophrenia & AD		schizophrenia & FTD		schizophrenia & PD	
	overlap (95% CI)	P value	overlap (95% CI)	P value	overlap (95% CI)	P value
Adipose_Subcutaneous	0.14 (0.03, 0.26)	2.23E-01	0.31 (0.19, 0.44)	1.63E-02	-0.05 (-0.18, 0.07)	6.58E-01
Adipose_Visceral_Omentum	0.06 (-0.11, 0.23)	7.21E-01	0.31 (0.14, 0.48)	8.00E-02	0.18 (0.04, 0.32)	1.95E-01
Adrenal_Gland	0.24 (0.03, 0.45)	2.50E-01	-0.16 (-0.34, 0.03)	3.97E-01	0.00 (-0.14, 0.15)	9.78E-01
Artery_Aorta	0.00 (-0.15, 0.15)	9.98E-01	0.20 (0.06, 0.34)	1.58E-01	-0.06 (-0.19, 0.07)	6.60E-01
Artery_Coronary	0.36 (0.21, 0.51)	2.17E-02	0.38 (0.14, 0.63)	1.31E-01	-0.09 (-0.26, 0.08)	6.14E-01
Artery_Tibial	-0.08 (-0.21, 0.05)	5.38E-01	0.26 (0.12, 0.40)	6.19E-02	0.01 (-0.11, 0.14)	9.15E-01
Brain_Amygdala	0.19 (-0.02, 0.41)	3.83E-01	0.25 (-0.01, 0.52)	3.48E-01	0.26 (0.07, 0.44)	1.72E-01
Brain_Anterior_cingulate_cortex_BA24	0.40 (0.23, 0.56)	2.29E-02	0.33 (0.14, 0.51)	8.54E-02	0.22 (0.07, 0.38)	1.66E-01
Brain_Caudate_basal_ganglia	0.21 (0.04, 0.38)	2.16E-01	-0.02 (-0.21, 0.16)	8.97E-01	0.02 (-0.14, 0.18)	9.01E-01
Brain_Cerebellar_Hemisphere	0.11 (-0.06, 0.29)	5.20E-01	-0.07 (-0.26, 0.13)	7.37E-01	-0.11 (-0.28, 0.07)	5.54E-01
Brain_Cerebellum	0.12 (-0.03, 0.28)	4.34E-01	0.19 (0.02, 0.37)	2.68E-01	-0.12 (-0.25, 0.01)	3.40E-01
Brain_Cortex	-0.03 (-0.22, 0.16)	8.68E-01	0.19 (0.00, 0.38)	3.21E-01	0.04 (-0.12, 0.20)	8.03E-01
Brain_Frontal_Cortex_BA9	0.32 (0.14, 0.50)	8.72E-02	0.16 (-0.05, 0.36)	4.50E-01	0.06 (-0.10, 0.22)	6.91E-01
Brain_Hippocampus	0.25 (0.07, 0.42)	1.72E-01	0.20 (-0.02, 0.43)	3.78E-01	-0.09 (-0.31, 0.12)	6.75E-01
Brain_Hypothalamus	0.48 (0.30, 0.66)	1.12E-02	0.13 (-0.20, 0.47)	6.94E-01	0.10 (-0.14, 0.34)	6.90E-01
Brain_Nucleus_accumbens_basal_ganglia	0.20 (0.04, 0.36)	2.18E-01	-0.01 (-0.19, 0.17)	9.66E-01	-0.02 (-0.17, 0.14)	9.23E-01
Brain_Putamen_basal_ganglia	0.15 (-0.02, 0.33)	3.92E-01	0.08 (-0.19, 0.35)	7.75E-01	0.25 (0.10, 0.41)	1.15E-01
Breast_Mammary_Tissue	0.23 (0.09, 0.37)	1.09E-01	0.32 (0.16, 0.47)	4.45E-02	0.08 (-0.10, 0.26)	6.63E-01
Cells_EBV-transformed_lymphocytes	0.29 (0.10, 0.48)	1.34E-01	0.24 (-0.03, 0.50)	3.79E-01	0.02 (-0.17, 0.22)	9.16E-01
Cells_Transformed_fibroblasts	0.22 (0.07, 0.37)	1.45E-01	0.13 (0.00, 0.26)	3.14E-01	-0.04 (-0.16, 0.08)	7.40E-01
Colon_Sigmoid	0.19 (0.04, 0.35)	2.07E-01	0.22 (0.05, 0.39)	1.93E-01	-0.17 (-0.31, -0.04)	2.13E-01
Colon_Transverse	0.24 (0.08, 0.39)	1.29E-01	0.27 (0.12, 0.43)	8.47E-02	0.10 (-0.04, 0.23)	4.78E-01
Esophagus_Gastroesophageal_Junction	0.22 (0.06, 0.38)	1.70E-01	0.31 (0.13, 0.48)	8.41E-02	-0.08 (-0.22, 0.06)	5.82E-01
Esophagus_Mucosa	0.09 (-0.04, 0.23)	5.02E-01	0.17 (0.02, 0.32)	2.60E-01	0.08 (-0.03, 0.20)	4.81E-01
Esophagus_Muscularis	-0.01 (-0.15, 0.13)	9.51E-01	0.20 (0.04, 0.36)	2.15E-01	0.04 (-0.08, 0.17)	7.32E-01
Heart_Atrial_Appendage	0.26 (0.11, 0.40)	8.72E-02	0.38 (0.25, 0.52)	6.29E-03	-0.07 (-0.20, 0.06)	5.92E-01
Heart_Left_Ventricle	0.26 (0.11, 0.41)	9.16E-02	0.17 (-0.04, 0.39)	4.17E-01	0.01 (-0.13, 0.16)	9.31E-01
Liver	0.25 (0.07, 0.44)	1.84E-01	0.53 (0.38, 0.69)	2.35E-03	-0.09 (-0.28, 0.09)	6.13E-01
Lung	0.19 (0.05, 0.32)	1.74E-01	0.36 (0.23, 0.48)	5.69E-03	-0.05 (-0.18, 0.08)	6.94E-01
Muscle_Skeletal	0.06 (-0.07, 0.19)	6.55E-01	**0.46 (0.33, 0.59)**	**6.81E-04**	-0.05 (-0.18, 0.08)	7.14E-01
Nerve_Tibial	0.24 (0.13, 0.35)	2.59E-02	-0.12 (-0.27, 0.02)	3.97E-01	-0.09 (-0.20, 0.02)	4.29E-01
Ovary	0.14 (-0.07, 0.36)	5.03E-01	-0.25 (-0.49, -0.02)	2.92E-01	0.18 (0.01, 0.35)	2.96E-01

GTEx tissue	schizophrenia & AD		schizophrenia & FTD		schizophrenia & PD	
	**overlap (95% CI)**	**P value**	**overlap (95% CI)**	**P value**	**overlap (95% CI)**	**P value**
Pancreas	0.12 (-0.05, 0.29)	4.67E-01	0.21 (0.02, 0.39)	2.67E-01	0.09 (-0.07, 0.24)	5.73E-01
Pituitary	0.23 (0.06, 0.39)	1.88E-01	0.36 (0.22, 0.49)	1.20E-02	-0.07 (-0.24, 0.09)	6.70E-01
Prostate	0.22 (0.04, 0.40)	2.25E-01	0.18 (0.00, 0.37)	3.29E-01	0.02 (-0.15, 0.18)	9.15E-01
Skin_Not_Sun_Exposed_Suprapubic	-0.03 (-0.17, 0.12)	8.40E-01	0.26 (0.13, 0.40)	5.82E-02	-0.09 (-0.22, 0.03)	4.71E-01
Skin_Sun_Exposed_Lower_leg	0.04 (-0.09, 0.17)	7.63E-01	0.35 (0.23, 0.48)	5.92E-03	-0.07 (-0.20, 0.05)	5.41E-01
Small_Intestine_Terminal_Ileum	0.19 (-0.10, 0.49)	5.19E-01	0.26 (0.07, 0.46)	1.79E-01	-0.17 (-0.35, 0.00)	3.34E-01
Spleen	0.03 (-0.12, 0.18)	8.45E-01	0.34 (0.15, 0.52)	7.72E-02	0.08 (-0.07, 0.24)	6.01E-01
Stomach	-0.07 (-0.25, 0.12)	7.11E-01	0.04 (-0.17, 0.25)	8.61E-01	-0.23 (-0.37, -0.08)	1.19E-01
Testis	0.11 (-0.01, 0.24)	3.42E-01	0.36 (0.24, 0.49)	5.10E-03	0.04 (-0.09, 0.17)	7.55E-01
Thyroid	0.21 (0.09, 0.32)	8.03E-02	0.29 (0.15, 0.42)	3.94E-02	0.07 (-0.03, 0.18)	4.97E-01
Uterus	0.37 (0.16, 0.59)	9.43E-02	-0.07 (-0.38, 0.23)	8.09E-01	-0.05 (-0.30, 0.21)	8.58E-01
Whole_Blood	0.18 (0.04, 0.32)	2.09E-01	0.32 (0.16, 0.49)	5.43E-02	0.08 (-0.07, 0.23)	6.01E-01

AD, Alzheimer's disease; PD, Parkinson's disease; FTD, frontotemporal dementia. P values in bold denote significant associations after the Bonferroni correction.

Supplementary Table 3. Cross-tissue Gene-disease associations for ALS and schizophrenia

Gene	ALS		schizophrenia	
	Test metric	P value	Test metric	P value
ZNHIT3	8.80	1.12E-04	8.30	1.90E-04
GLB1L3	7.17	8.64E-04	11.46	9.80E-06
TMEM194A	6.99	3.83E-04	7.14	3.30E-04

Supplementary Figure 1. LDSC-SEG analysis of GWAS enrichment in mouse CNS cells

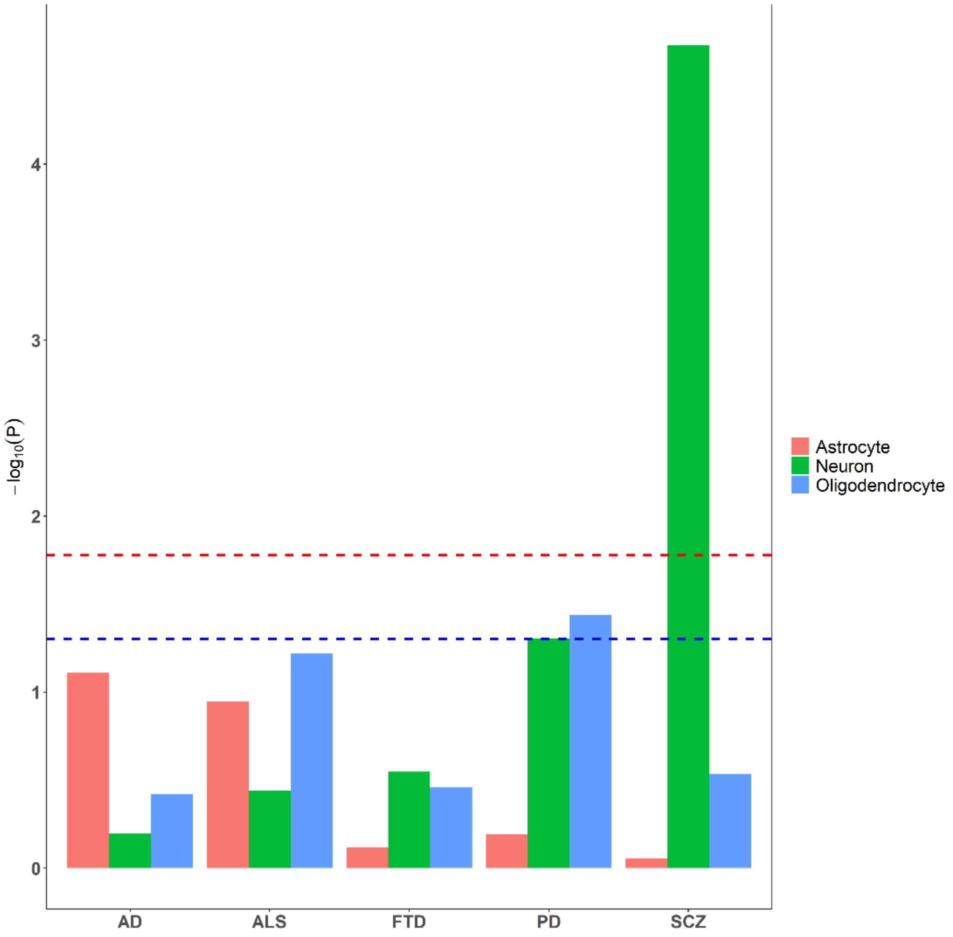

The red dashed line demarks the multiple-test corrected threshold (P=0.05/3), while the blue dotted line demarks the nominal threshold (P = 0.05).

Supplementary Figure 2. Tissue-specific disease-inferred gene expression profile correlation between schizophrenia and AD.

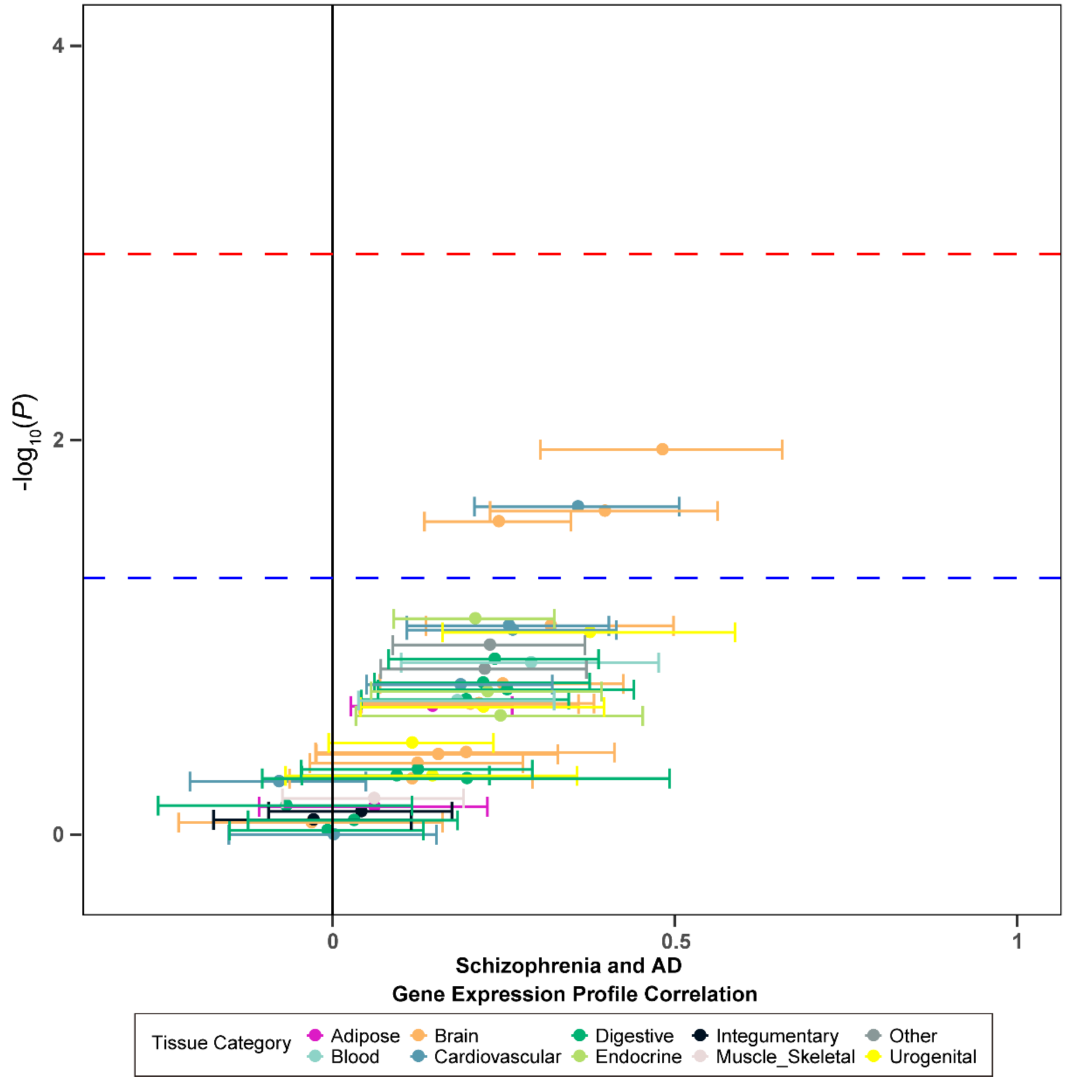

The red dashed line demarks the multiple-test corrected threshold (P=0.05/48), while the blue dotted line demarks the nominal threshold (P = 0.05).

Supplementary Figure 3. Tissue-specific disease-inferred gene expression profile correlation between schizophrenia and FTD.

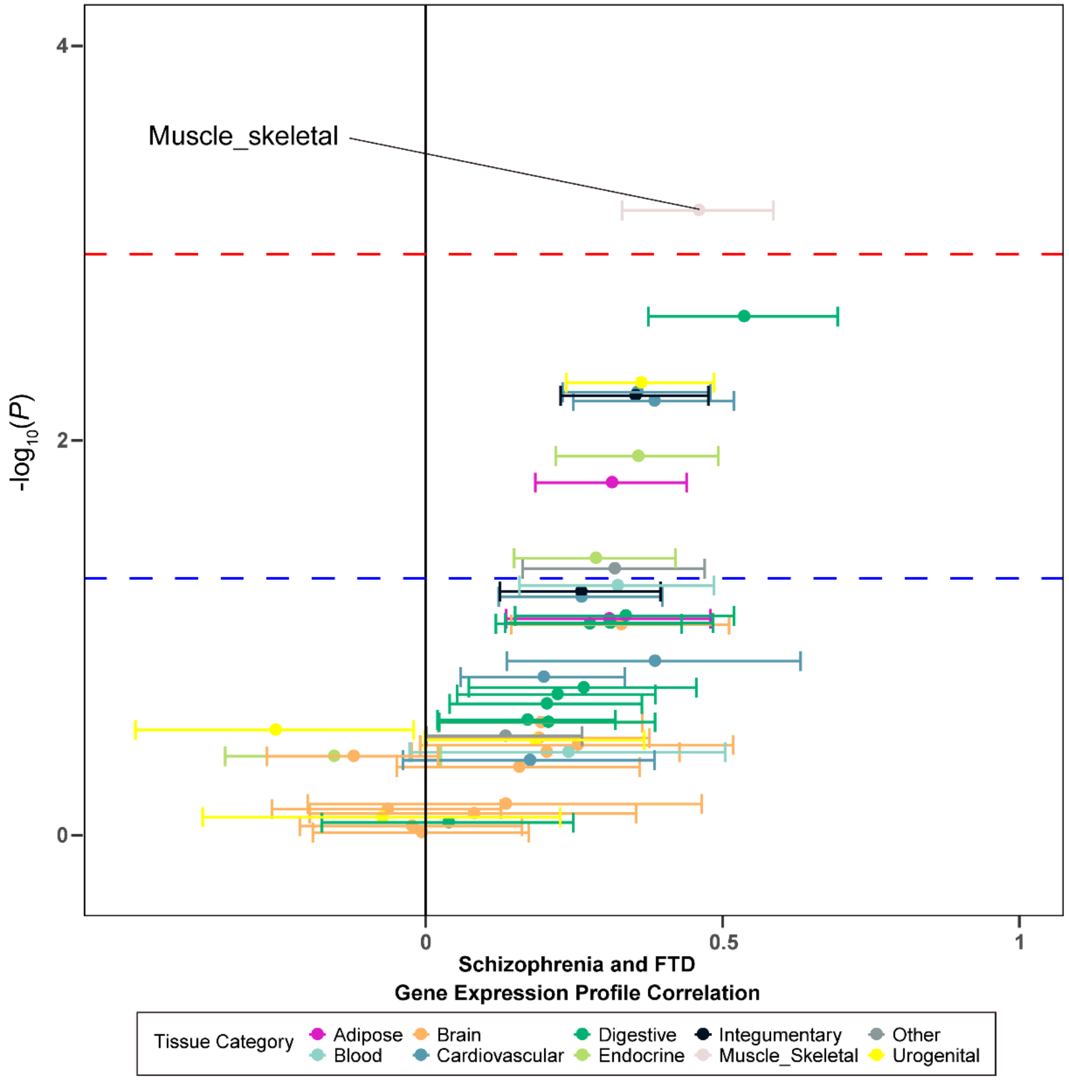

The red dashed line demarks the multiple-test corrected threshold (P=0.05/48), while the blue dotted line demarks the nominal threshold (P = 0.05).

Supplementary Figure 4. Tissue-specific disease-inferred gene expression profile correlation between schizophrenia and PD.

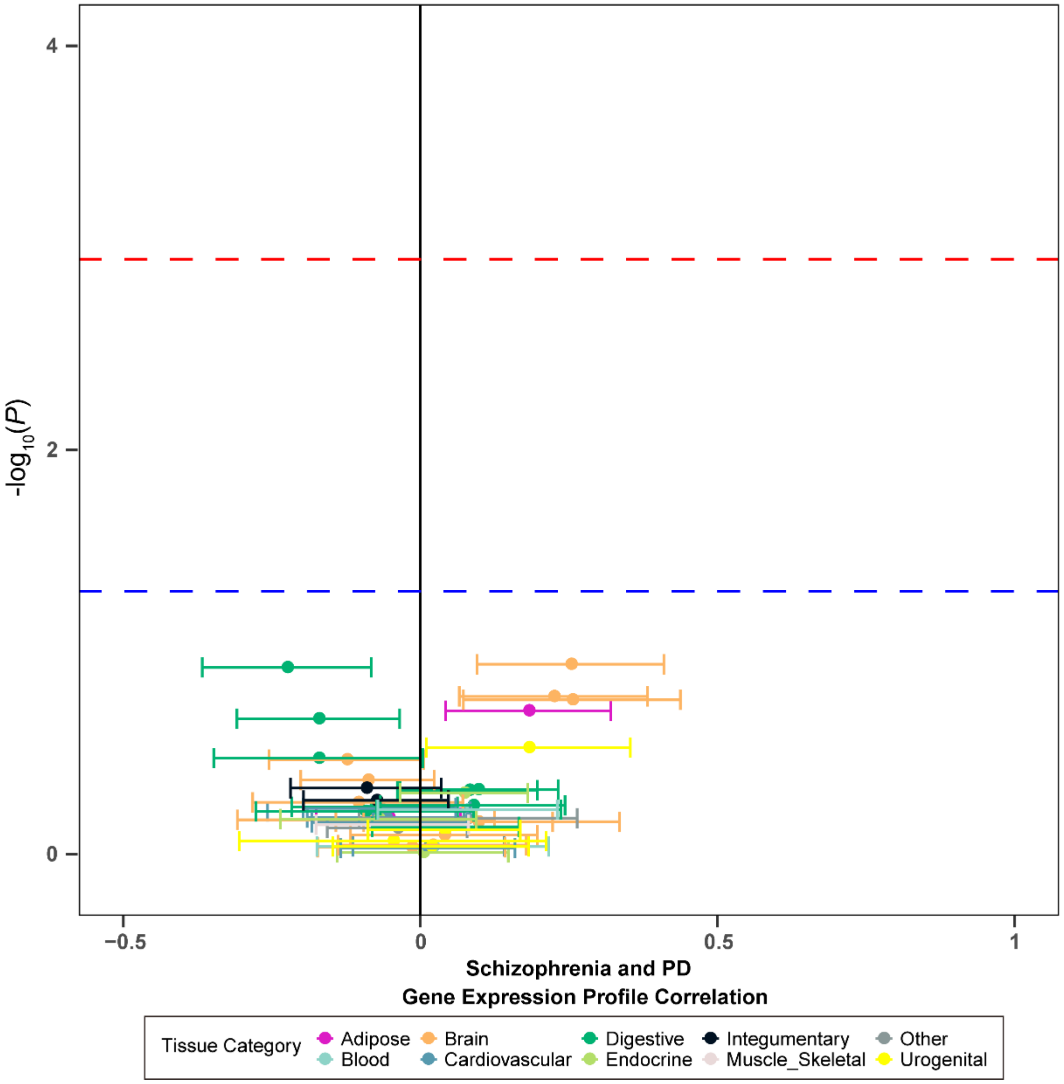

The red dashed line demarks the multiple-test corrected threshold (P=0.05/48), while the blue dotted line demarks the nominal threshold (P = 0.05).

Supplementary Figure 5. Manhattan-style plots showing the estimates of local SNP-heritability of schizophrenia and PD.

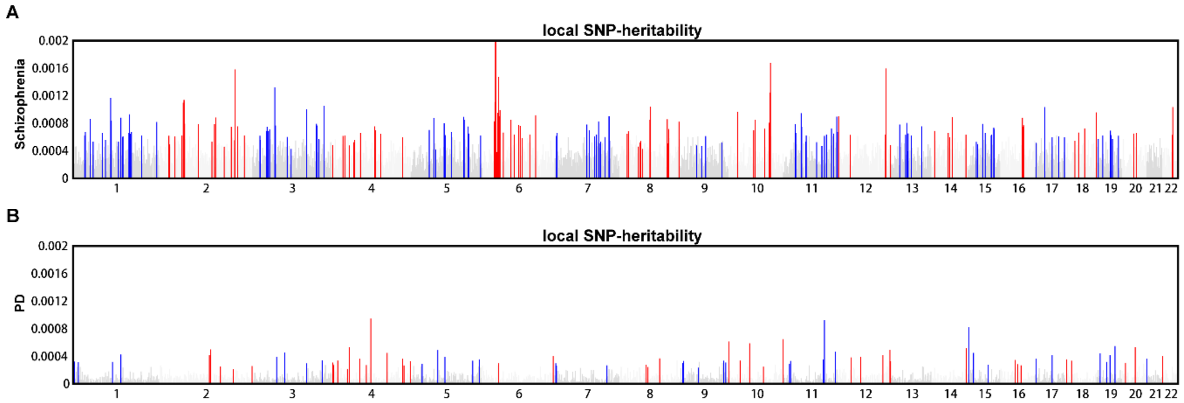

PD, Parkinson's disease.
